# Comparison of Ultra-Rapid Orbit Prediction Strategies for GPS, GLONASS, Galileo and BeiDou

**DOI:** 10.3390/s18020477

**Published:** 2018-02-06

**Authors:** Tao Geng, Peng Zhang, Wei Wang, Xin Xie

**Affiliations:** 1 GNSS Research Center, Wuhan University, No.129 Luoyu Road, Wuhan 430079, China; gt_gengtao@whu.edu.cn (T.G.); xiexin@whu.edu.cn (X.X.); 2 Collaborative Innovation Center of Geospatial Technology, No.129 Luoyu Road, Wuhan 430079, China; 3 Beijing Institute of Tracking and Telecommunication Technology, No. 26 Beiqing Road, Beijing 100094, China

**Keywords:** predicted orbits, ultra-rapid, GNSS, solar radiation pressure, IGS, MGEX

## Abstract

Currently, ultra-rapid orbits play an important role in the high-speed development of global navigation satellite system (GNSS) real-time applications. This contribution focuses on the impact of the fitting arc length of observed orbits and solar radiation pressure (SRP) on the orbit prediction performance for GPS, GLONASS, Galileo and BeiDou. One full year’s precise ephemerides during 2015 were used as fitted observed orbits and then as references to be compared with predicted orbits, together with known earth rotation parameters. The full nine-parameter Empirical Center for Orbit Determination in Europe (CODE) Orbit Model (ECOM) and its reduced version were chosen in our study. The arc lengths of observed fitted orbits that showed the smallest weighted root mean squares (WRMSs) and medians of the orbit differences after a Helmert transformation fell between 40 and 45 h for GPS and GLONASS and between 42 and 48 h for Galileo, while the WRMS values and medians become flat after a 42 h arc length for BeiDou. The stability of the Helmert transformation and SRP parameters also confirmed the similar optimal arc lengths. The range around 42–45 h is suggested to be the optimal arc length interval of the fitted observed orbits for the multi-GNSS joint solution of ultra-rapid orbits.

## 1. Introduction

Since the release of the International Global Navigation Satellite System (GNSS) Service (IGS, [1]) ultra-rapid (IGU) products, real-time and near-real-time GNSS applications have undergone unprecedented development, for example, in real-time precise point positioning (PPP), tropospheric and ionospheric monitoring, real-time clock estimation, and rapid orbit determination for low earth-orbiting satellites [2,3,4,5,6,7].

In April 2000, IGS began to provide subdaily IGU–GPS combined orbit products to the world at 00:00 and 12:00 UTC each day. Since GPS week 1267, IGS shortened the update cycle to 6 h by adding 06:00 and 18:00 UTC. Each IGU orbit file contains the observed orbits for the first 24 h and the predicted orbits for the next 24 h [8]. Once the IGS rapid (IGR) products are released every day, they are used as references to compare with the IGU combination orbits for the full observed orbits and the first 6 h of predicted orbits. The weighted root-mean-square (WRMS) and median orbit residuals usually fall between 5 and 10 mm for IGU combined GPS observed orbits and between 15 and 20 mm for the first 6 h of recent predicted orbits. (See http://www.igs.org/analysis/gps-ultra) To develop GPS + GLONASS near real-time zenith troposphere delay solutions, the Geodetic Observatory Pecný (GOP) started to produce ultra-rapid GLONASS orbit products in 2009. In addition, the products they have developed have achieved an overall accuracy of 10 cm for the full observed orbits and 15–30 cm for 12 h predicted orbits [9]. The IGU GLONASS combination orbit (IGV) started testing on 9 September 2010 and became a new official product in 2014 after 4 years of experiments. Each IGV SP3 file contains GPS orbits, clocks and GLONASS orbits only for 24 h observations and 24 h predictions [10]. Currently, several analysis centers (ACs), for example, the Center for Orbit Determination in Europe (CODE), Natural Resources Canada (NRC), the European Space Agency (ESA), GeoForschungsZentrum (GFZ), GOP and Wuhan University (WHU), have been contributing to IGU combinations. The WRMS and median orbit residuals of each individual AC’s orbits with respect to the IGS ultra-rapid GLONASS combination orbits over the entire 48 h can achieve an accuracy of 20–100 mm (see http://acc.igs.org/ analysis/glonass-ultra).

Assuming that x=(r,v,p), then the differential equation and initial condition of the satellite motion can be written as

(1) $$\{\begin{array}{c} \dot{x}=F\left(x,t\right)\\ x{|}_{t_{0}}=x_{0} \end{array}$$

where x is the condition at any time of the satellite motion including position *r*, velocity *v* and dynamical parameters *p*; *F(x, t)* is the satellite’s perturbation equation; and $t_{0}$ is the initial time. For a given dynamic model, the position and velocity of a satellite at any time can be obtained by numerical integration. In the process of ultra-rapid orbit prediction, the precise initial condition is obtained by the observed orbits. Thus, the most important parts of the prediction strategy are the arc length of observed orbits and which dynamic model should be used to generate the precise predicted orbits.

By using known earth rotation parameters (ERPs) and IGS rapid orbits as “truth”, Choi et al. adopted an almost ideal situation to study the effects of fitting the arc length of observed orbits and the parameterization strategy used to estimate the solar radiation pressure (SRP) on GPS predicted orbits’ performance. They concluded that the optimal arc length of observed orbits for fitting is around 40–45 h, together with using the CODE SRP model [11]. Moreover, Li et al. obtained a similar conclusion for GPS: that a better orbit prediction performance can be obtained by using an arc length for fitting orbits of around 40 h; they also studied the relationship between SRP parameters and the accuracy of GPS predicted orbits [12].

After the full deployment of US GPS and Russian GLONASS, the Chinese BeiDou Navigation Satellite System (BDS) and the European Galileo Navigation Satellite System (Galileo) are now under construction and are being gradually improved. Until December 2015, the BDS constellation had included five geostationary earth orbit (GEO), five inclined geo-synchronous orbit (IGSO) and three medium earth orbit (MEO) satellites providing on-orbit services. BDS is now in the period of rapid deployment towards global service. The space constellation of BDS will consist of 5 GEO satellites, 27 MEO satellites and 3 IGSO satellites when fully deployed [13]. As for Galileo, the first four in-orbit validation (IOV) satellites have completed the test phase and have been in orbit. At present, the IOV phase has been closed, and it is in the period of turning to the full operational capability (FOC) stage (see more details in Table 1). Multi-GNSS, GPS, GLONASS, BDS and Galileo provide much scientific research with the increase in satellite numbers and the improvement of the PDOP values to achieve higher accuracy and stability [5,6,14]. Unfortunately, compared to the rapid development of multi-GNSS real-time applications, there is little research on the predicted orbits of GLONASS, Galileo and BDS.

This contribution focuses on the impact of the fitting arc length of observed orbits and SRP on the orbit prediction performance for GPS, GLONASS, Galileo and BeiDou. In Section 2, the model for generating predicted orbits and statistical methods in our study are described. The data collection and dynamic model used for orbit prediction are summarized in Section 3. The results of the performance of predicted orbits are shown in Section 4. Finally, conclusions are summarized in the last section.

## 2. Basic Model

Among the processes of orbit prediction, the prediction errors of the earth orientation parameters (EOPs) cannot be ignored. The impact of EOP errors on the orbit prediction has been studied in many researches. As for satellite orbits extrapolated by using the GPS precise ephemerides, the orbital error caused by EOP prediction approximates 0.232 ± 0.183 m in one day, and the error caused by UT1 is more than that of polar motion [15,16]. As the precession and nutation can be accurately determined and because the accuracy of the predicted model is high, the EOPs’ predicting work is mainly focused on the high-precision prediction of polar motion and UT1-UTC, the ERPs. To prevent the influence of ERP prediction errors, the ERP parameters from IERS Bulletin A (see https://www.iers.org/IERS/EN/DataProducts/EarthOrientationData/eop.html) were used as the truth in our study.

We computed orbit predictions for 24 h in the future, using final precise orbit products extending *N* hours in the past, for *N* = 24 through 72, in 1 h increments. These final precise orbits are also called “observed orbits” in this contribution. Using the known ERPs, 49 fitted observed orbits were used as pseudo-measurements, were rotated from the earth-fixed frame to an inertial frame and then were fitted to the chosen orbit dynamic model with integration to obtain the precise initial conditions, which included the satellites’ initial states and estimated SRP parameters. The next 24 h predicted orbits in the future were obtained through orbit integration by using the precise initial conditions.

**Figure 1:**
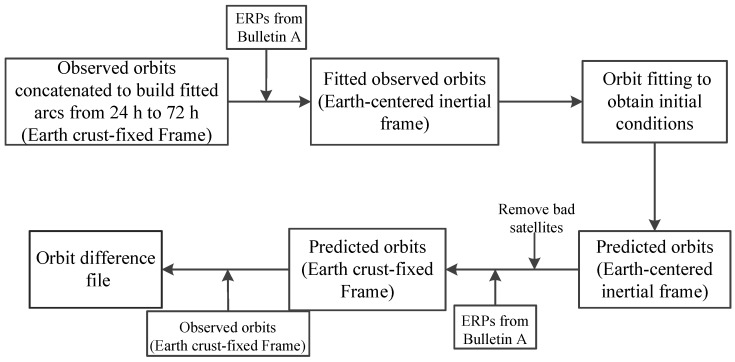
Workflow of generating predicted orbits.

After that, the 24 h predicted orbits were rotated back to earth-fixed coordinates. Besides the quasi-random subdaily WRMS scatter, the orbit products also contained the error of possible systematic frame difference [11]. In order to better analyze the impact of the fitting arc length of observed orbits and SRP on the orbit prediction performance for a quad-system, we used the Helmert transformation (Equation (2)) to separate the systemic frame error:

(2) $$\left[\begin{array}{c} X_{2}\\ Y_{2}\\ Z_{2} \end{array}\right]=\left[\begin{array}{c} T_{X}\\ T_{Y}\\ T_{Z} \end{array}\right]+\left(1+M\right)\cdot \left[\begin{array}{ccc} 1 & R_{Z} & -R_{Y}\\ -R_{Z} & 1 & R_{X}\\ R_{Y} & -R_{X} & 1 \end{array}\right]\left[\begin{array}{c} X_{1}\\ Y_{1}\\ Z_{1} \end{array}\right]$$

where *M* is an orbital frame scale factor, $T={\left[\begin{array}{ccc} T_{X} & T_{Y} & T_{Z} \end{array}\right]}^{T}$ is a translational vector with respect to the orbital origin, and $R={\left[\begin{array}{ccc} R_{X} & R_{Y} & R_{Z} \end{array}\right]}^{T}$ is a rotational vector with respect to each coordinate axis. The stability of these Helmert transformation parameters was used as an additional indication of the orbit prediction performance. The Position and Navigation Data Analyst (PANDA) software developed by GNSS research center of Wuhan University in China [17] was used for all the data processing of the orbit predictions in our study. The workflow is shown in Figure 1.

The WRMS and median orbit residuals of orbit differences were used to evaluate the accuracy of the first 6 h and full 24 h of predicted orbits. The spherical standard error (SSE) was computed for every satellite using Equation (3) [11]:

(3) $$SSE_{i}=\frac{\sigma_{X}^{i}+\sigma_{Y}^{i}+\sigma_{Z}^{i}}{3}$$

where $\sigma_{X,Y,Z}$ are the standard deviations (STDs) of the orbit differences and i is the satellite. Assuming that the mean of the orbit differences was zero, we weighted the SSE of each satellite to obtain the WRMS of an entire constellation:

(4) $$WRMS=\frac{\sum_{i=1}^{n}\left(\omega_{i}SSE_{i}\right)}{\sum_{i=1}^{n}\omega_{i}}$$

where $\omega_{i}$ represents the weight of each satellite and can be calculated by the following formula:

(5) $$\omega_{i}=\frac{1}{2^{\alpha_{i}}}$$

where α is the orbit accuracy exponent from the precise ephemerides header.

## 3. Data Processing

### 3.1. Data Collection

This paper adopted year-round data from 2015. IGS rapid orbits were used for the study of GPS. As a result of the IGS not having released GLONASS rapid orbits, the IGS final orbits were used for GLONASS [18]. Because there were no combined orbit products in the Multi-GNSS EXperiment (MGEX) [19] for Galileo and BeiDou, the final orbit products from the GNSS Research Center of WHU, China were used in our study [20]. Not only as fitting data, these orbit products were also used as references to assess the accuracy of the predicted orbits. GPS satellites with “Notice Advisory to Navstar Users” advisories were excluded from consideration during their announced outages or unavailability; the same applied for GLONASS with “Notice Advisory to GLONASS Users” and Galileo with “Notice Advisory to Galileo Users”. As for BDS, we did not find any official notice for users; thus the satellites whose three dimensional (3D) RMS of orbit fitting residuals were greater than 1 m were removed. BeiDou-GEO was not within our consideration because of its larger unmodeled orbital errors [21].

### 3.2. Dynamic Model

As the navigation satellite’s orbit altitude is usually around 20,000 km, compared with other nonconservative forces, direct SRP produces a greater perturbation. On the basis of the optical properties of the surface materials and the structure information of satellites and long-term GNSS tracking data, three types of SRP models have been proposed and used for GNSS: (1) Analytical models, for example, ROCK models [22,23] and the UCL (University College London) model [24]. The main shortcoming of the analytical models is that they must be provided with accurate parameters of the structure and optical properties of the satellites and that every error in the parameters will result in orbital error; (2) Empirical models, for example, the Empirical (CODE) Orbit Model (ECOM) and its reduced or extended versions [25,26,27]. These models are built on real long-term GNSS tracking data. Although they do not reflect the true physical significance of satellites in orbit, they are able to effectively compensate for the perturbation effects of SRP, and it is easy to modify the orbit dynamic model; (3) Semi-analytical and -empirical models, for example, the adjustable box-wing model [28] and GPS Solar Pressure Model (GSPM; [29,30]). These models can be of the same true physical significance as the analytical models and of the same orbit accuracy as the empirical models.

From the precise orbit determination strategy adopted by ACs in the IGS or MGEX (see ftp://ftp.igs.org/pub/center/analysis and [21]), we can clearly find that most of the ACs use the full nine-parameter ECOM or its reduced version. Therefore, we used the ECOM and its reduced version in our orbit prediction strategy in order to be consistent with most of the IGS ACs. The full nine-parameter ECOM is established in a sun–earth oriented frame with directions denoted by *DYB* and nine empirical parameters estimated:

(6) $$\begin{array}{c} \alpha_{srp,D}=D_{0}+D_{c}\cos u+D_{s}\sin u\\ \alpha_{srp,Y}=Y_{0}+Y_{c}\cos u+Y_{s}\sin u\\ \alpha_{srp,B}=B_{0}+B_{c}\cos u+B_{s}\sin u \end{array}$$

where the *D* direction is perpendicular to the solar panel and points to the sun in inertial space, the *Y* direction points along the solar panel beams, and the *B* direction constitutes the right-handed system with the other two directions. ${\left[D_{0}\;Y_{0}\;B_{0}\right]}^{T}$, ${\left[D_{c}\;Y_{c}\;B_{c}\right]}^{T}$ and ${\left[D_{s}\;Y_{s}\;B_{s}\right]}^{T}$ represent the constant and once-per-revolution sine and cosine terms, respectively, and *u* represents the argument of latitude of the satellite. However, because some of the nine parameters may weaken the orbital solutions [26], the model’s reduced version is mostly used in practice, only estimating the constant and periodic terms in the *B* direction and the constant terms in the *D* and *Y* directions. Hereafter, we denote this reduced version as ECOM-5 and the full nine-parameter ECOM as ECOM-9.

During 2015, most of the IGS ACs added earth radiation correction in their strategies for determining the orbits of GPS and GLONASS. We considered a globally constant albedo of *α* = 0.3 for the earth radiation model together with the analytical box-wing model [31]; see Table 2 for further details.

## 4. Results and Discussion

It is necessary to point out that GPS Block-IIA, BeiDou-IGSO and -MEO satellites cause special modeling problems. During eclipse seasons, GPS Block-IIA satellites perform shadow and post-shadow yaw maneuvers. Thus Block-IIA satellites show a lower performance during eclipses than Block-IIR satellites, for which the yaw attitude is simple to model and predict (very close to nominal yaw-steering (YS) attitude in most case) [36]. BeiDou-IGSO and -MEO satellites adopted the attitude mode of switching between YS and orbit-normal (ON) modes [37]. The yaw attitude switches from the YS mode to the ON mode, and vice versa, when the latitude of the satellite equals 90° and the elevation angle of the sun above the orbital plane is close to 4° [38]. During the ON mode period, significant accuracy degradation of predicted orbits has been shown in our study.

**Figure 2:**
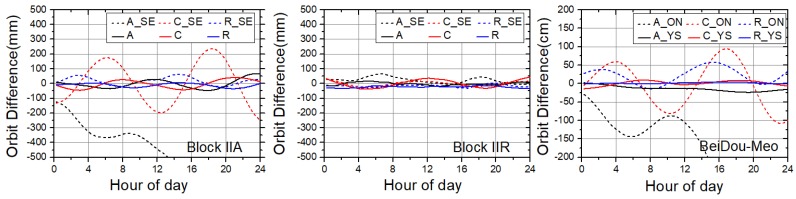
Orbit differences between predicted orbits and precise ephemerides in inertial space after Helmert frame transformation for Block-IIA (G32, **left**), Block-IIR (G11, **middle**) and BeiDou-MEO (medium earth orbit) (C12, **right**) satellites. A, C and R represent along-track, cross-track and radial direction on solar visible day, respectively. A_SE, C_SE and R_SE represent along-track, cross-track and radial direction on solar eclipse day, respectively. ON and YS represent orbit-normal and yaw-steering modes, respectively.

The orbit differences between our predicted orbits and the precise ephemerides after a Helmert transformation in a body-fixed frame are illustrated in Figure 2. The panel on the left shows the orbit differences of the Block-IIA satellite (G32) on a solar eclipse day (8 October 8) and a solar visible day (21 January), and the middle panel shows those for the Block-IIR satellite (G11; January, in solar eclipse season; 21 January, in solar visible season). It can be clearly seen that great fluctuations and divergence occurred during the solar eclipse season for the Block-IIA satellites. However, the Block-IIR satellite continued its good performance, particularly in the along-track direction. In the panel on the right, the orbit differences between predicted orbits and precise ephemerides for the BeiDou-MEO satellite (C12) in ON mode (1 January) and YS mode (1 February) are shown. The bad performance is clearly shown in the ON mode period.

**Figure 3:**
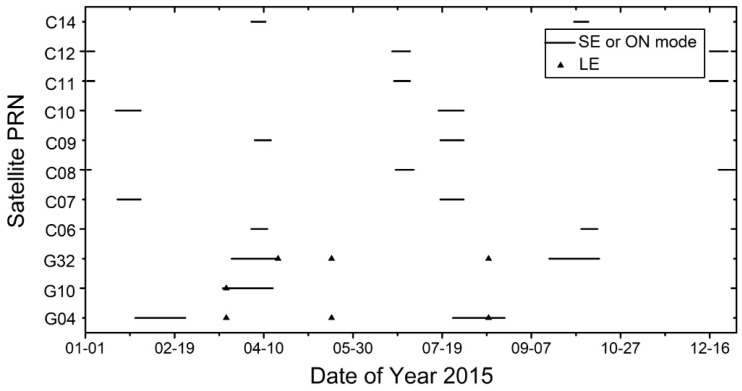
Data deleted from Block-IIA (G04, G10 and G32) in eclipse season and BeiDou Navigation Satellite System (BDS) satellites in orbit-normal (ON) mode (SE represents solar eclipse and LE represents lunar eclipse).

In order to avoid the impact of the above situation on the final results, the data of Block-IIA in the solar and lunar eclipse seasons as well as BeiDou-MEO/IGSO satellites in ON mode were excluded from our study (bad satellites removed; Figure 1). The deleted data are plotted in Figure 3.

### 4.1. Orbit Differences

**Figure 4:**
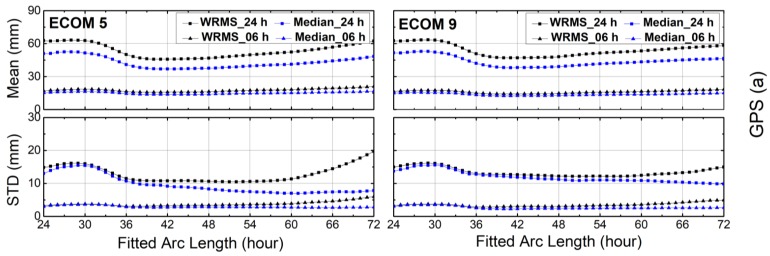
The means and standard deviations (STDs) of weighted root-mean-square (WRMS) and median residuals for orbit differences between predicted orbits and precise ephemerides after Helmert frame transformation over the first 6 h and full 24 h intervals for GPS. Results for reduced Empirical Center for Orbit Determination in Europe Orbit Model (ECOM-5) are shown on the left and for nine-parameter (ECOM-9) on the right planes.

**Figure 5:**
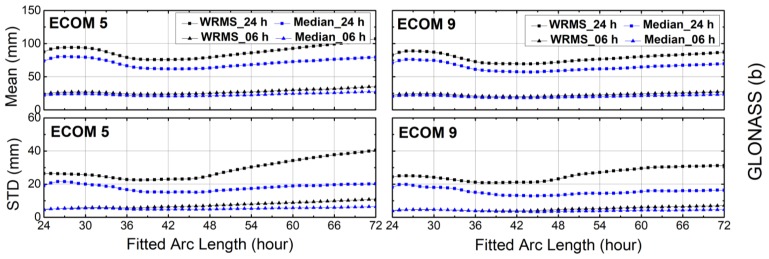
The means and standard deviations (STDs) of weighted root-mean-square (WRMS) and median residuals for orbit differences between predicted orbits and precise ephemerides after Helmert frame transformation over the first 6 h and full 24 h intervals for GLONASS. Results for reduced Empirical Center for Orbit Determination in Europe Orbit Model (ECOM-5) are shown on the left and for nine-parameter (ECOM-9) on the right planes.

**Figure 6:**
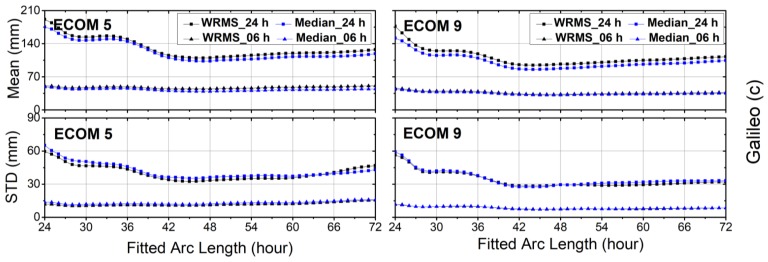
The means and standard deviations (STDs) of weighted root-mean-square (WRMS) and median residuals for orbit differences between predicted orbits and precise ephemerides after Helmert frame transformation over the first 6 h and full 24 h intervals for Galileo. Results for reduced Empirical Center for Orbit Determination in Europe Orbit Model (ECOM-5) are shown on the left and for nine-parameter (ECOM-9) on the right planes.

**Figure 7:**
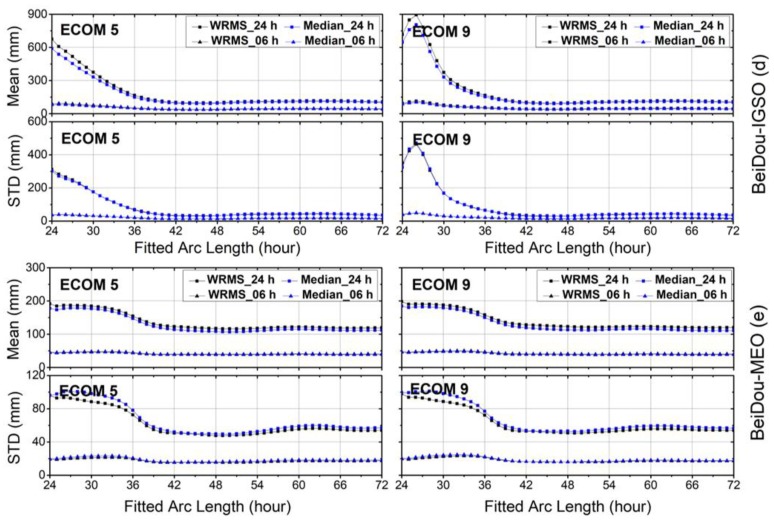
The means and standard deviations (STDs) of weighted root-mean-square (WRMS) and median residuals for orbit differences between predicted orbits and precise ephemerides after Helmert frame transformation over the first 6 h and full 24 h intervals for BeiDou inclined geo-synchronous orbit (IGSO)/medium earth orbit (MEO). Results for reduced Empirical Center for Orbit Determination in Europe Orbit Model (ECOM-5) are shown on the left and for nine-parameter (ECOM-9) on the right planes.

The WRMS values of the orbit differences after the Helmert transformation were computed by using Equations (2)–(5). The mean values and STDs of the WRMS and median for each fitted arc length for GPS, GLONASS, Galileo and BDS are plotted in Figure 4, Figure 5, Figure 6 and Figure 7. It is clear from Figure 4 that the fitted arc lengths that had the smallest WRMS and median values for GPS predicted orbits fell between 40 and 45 h over both SRP models, which was almost identical to results of Choi et al. [11]. However, the variation in STDs for the WRMS and median was more moderate, particularly for WRMS 24 h. The reason may be that there were more Block-IIA satellites in the year 2012 used by Choi et al. [11]. 

For other GNSSs, the arc lengths that showed the smallest WRMS and median values fell between 40 and 45 h for GLONASS, and between 42 and 48 h for Galileo (Figure 5 and Figure 6, respectively). The WRMS and median values during the above-selected arc length intervals showed very small differences for each navigation system. We can also see that the means and STDs of the WRMS and median for BeiDou-IGSO/MEO were quite flat after 42 h, particularly for BeiDou-IGSO (Figure 7). In addition, the curves of Galileo and BeiDou dropped sharply between 24 and 36 h of fitting the arc length. The reason could be that the orbit dynamic models, especially solar radiation pressure model of Galileo and BeiDou are not accurate enough compared to those of GPS and GLONASS. 

Compared with ECOM-9, the STD curves of ECOM-5 varied strongly with the arc length for 24 h predicted solutions of GPS, GLONASS and Galileo, while the curves of ECOM-5 and ECOM-9 showed similar trends for BeiDou-MEO. As for BeiDou-IGSO (Figure 7), the curves of ECOM-9 have cusps between 24 and 28 h for the 24 h predicted solutions, and the reason for this needs to be studied further.

The means and STDs of WRMS and median residuals in the selected fitted arc lengths for the four navigation satellite systems are shown in Table 3. As can be seen from Table 3, the WRMS and median of Galileo and BeiDou were around 10 cm for 24 h predictions, which were much larger than those of GPS and GLONASS, that is, because of the poorer accuracy of the observed orbits and the imperfect orbit model.

For GLONASS, the WRMS and median residuals of ECOM-9 were around 10% lower than for ECOM-5 in the 40–45 h fitted interval for 6 and 24 h predictions. For Galileo, the values of ECOM-9 were around 20% lower than for ECOM-5 in the 42–48 h fitted interval. However, the differences between ECOM-5 and ECOM-9 were not significant for GPS or BeiDou. 

### 4.2. Helmert Alignment

The means and STDs of the Helmert transformation parameters for the quad-system are plotted in Figures S1–S5 in the supporting information. For GPS, the optimal arc length for the stability of the Helmert parameters was selected as 40–45 h. Although some curves of the STDs did not show the minimum between 40 and 45 h, for example, $T_{Y}$-06 h and $T_{Z}$-06 h for both ECOM-5 and ECOM-9 (Figure S1), the difference in minimum values between the 24–72 and 40–45 h arc lengths was less than 0.5 mm. In addition, the STDs became larger for other arc lengths compared with those for 40–45 h, particularly for translational parameters.

On the whole, the Helmert transformation parameters of GLONASS and Galileo showed relatively better stability between 36–45 and 42–48 h, respectively, although $T_{Z}$-24 h and $T_{Z}$-06 h for both SRP models for GLONASS and $T_{Z}$-06 h for Galileo showed different trends (Figure S2). Moreover, the curves of BeiDou-IGSO/MEO became flat after 42 h, except the translational parameters of BeiDou-MEO, which showed some fluctuations likely due to a small satellite number.

The effects of the Helmert transformation parameters on orbits at the nominal altitude of each navigation system in the selected fitted arc length intervals are summarized in Tables S1–S5 in the supporting information. In fact, the scale and translation offsets had smaller impacts on the orbit accuracy to the rotational parameters [11]. Table 4 lists the results of the effects of the Hermert rotational parameters on the orbits. From Table 4, GPS and GLONASS showed more stable rotational parameters than Galileo, BeiDou-IGSO, and BeiDou-MEO. This may have been due to the poor observed orbits, small satellite numbers, and imperfect orbit models of Galileo and BeiDou.

### 4.3. Solar Radiation Pressure

**Figure 8:**
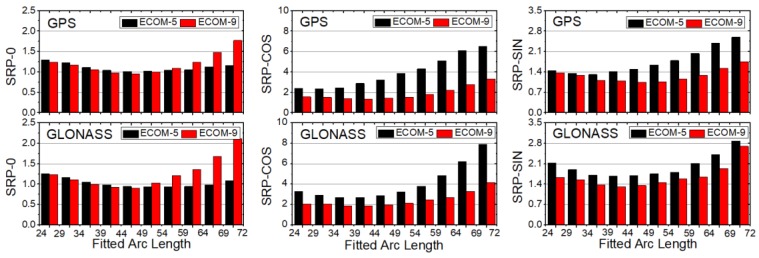
Root mean squares (RMSs) of daily variations in GPS and GLONASS solar radiation pressure (SRP) parameters estimated with various arc lengths over the year 2015 (SRP-0, -COS and -SIN represent the constant and once-per-revolution sine and cosine terms, respectively; unit: 10^−3^).

It is commonly acknowledged that SRP is difficult to model because of its complexity, and the unmodeled error will directly affect the precision of the orbit prediction. Thus, in order to figure out how the SRP parameter affects the accuracy of the prediction orbits, the stability of the SRP constant (SRP, 0) and the once-per-revolution sine (SRP, SIN) and cosine (SRP, COS) terms using Equation (7) is shown in Figure 8.

(7) $$\begin{array}{c} RMS_{SRP,0}=\frac{1}{3}\cdot \sum_{d=D,Y,B}^{}\sqrt{\frac{1}{m}\cdot \left(\sum_{isat=1}^{m}\frac{\sum_{i=2}^{n}{\left(SRP,0_{i}^{d}-SRP,0_{i-1}^{d}\right)}^{2}}{n-1}\right)}\\ RMS_{SRP,COS}=\frac{1}{3}\cdot \sum_{d=D,Y,B}^{}\sqrt{\frac{1}{m}\cdot \left(\sum_{isat=1}^{m}\frac{\sum_{i=2}^{n}{\left(SRP,COS_{i}^{d}-SRP,COS_{i-1}^{d}\right)}^{2}}{n-1}\right)}\\ RMS_{SRP,SIN}=\frac{1}{3}\cdot \sum_{d=D,Y,B}^{}\sqrt{\frac{1}{m}\cdot \left(\sum_{isat=1}^{m}\frac{\sum_{i=2}^{n}{\left(SRP,SIN_{i}^{d}-SRP,SIN_{i-1}^{d}\right)}^{2}}{n-1}\right)} \end{array}$$

where n represents the day of the year in 2015, m represents the satellite number, and d represents the direction of the *DYB* frame in Equation (5). Because Galileo and BDS are still under construction and there are no combined orbit products, these two navigation systems were not considered here.

As can be seen from Figure 8, the trends of SRP parameters for GPS were similar to those for GLONASS. The RMS values of daily variations showed similar “U”-shape patterns for different SRP parameters. For GPS, the arc lengths for ECOM-5 that showed the smallest RMS values fell in the 44–48, 29–33, and 34–38 h fitted intervals for the constant, cosine and sine terms, respectively, but the arc lengths fell between 44 and 48 h for all three SRP terms for ECOM-9. For GLONASS, the RMS values achieved a minimum in the 39–48 h fitted intervals for all three terms for both ECOM-5 and ECOM-9. In addition, for the cosine and sine terms, RMS values of ECOM-9 were smaller than those of ECOM-5 for GPS and GLONASS. For the constant term, the difference between ECOM-5 and ECOM-9 was very small before 48 h, but RMS values of ECOM-9 had stronger variation after 48 h.

## 5. Conclusions

This study has contributed to the focus on the impact of the fitting arc length of observed orbits and SRP on the orbit prediction performance for GPS, GLONASS, Galileo and BeiDou. The impacts are analyzed from three aspects, orbit differences, Helmert alignment and the stability of SRP parameters. As for orbit differences, the fitted arc lengths with the smallest WRMS and median values were 40–45, 40–45, 42–48, 40–50 and 42–54 h (Table 3) for GPS, GLONASS, Galileo, BeiDou-IGSO and BeiDou-MEO, respectively. As for the Helmert alignment parameters, the fitted arc lengths of 40–45, 36–45, 42–48, 42–48 and 39–54 h (Table 4) were more stable for each navigation satellite system, respectively. The results show that the performance of the predicted orbit was closely related to the arc lengths of fitted orbits and the stability of SRP parameters. In general, the fitted arc lengths of 40–48 and 44–48 h (Figure 8) had the smallest RMS values for the GPS and GLONASS SRP parameters. When considering the joint solution of multi-GNSS for ultra-rapid orbits in the IGS MGEX project, the optimal arc length interval is suggested as 42–45 h on the basis of the above analysis.

However, there are still many unsolved problems that need to be further explored. In the process of real orbit prediction, this will inevitably suffer from ERP prediction errors when coordinates convert between the earth-fixed reference frame and the earth-center inertial frame, as is discussed in Section 2. In most cases, the impact of ERP prediction errors is the main source of orbit prediction error [39]. Meanwhile, it should be mentioned that the stability of SRP parameters should be studied when the constellation is completed and the orbit’s combined products are released for Galileo and BDS. It is equally important to develop the appropriate SRP model for orbit prediction when Block-IIA satellites experience eclipse season and BeiDou-MEO/IGSO satellites are in ON mode.

## Figures and Tables

**Table 1:** BeiDou and Galileo satellites’ on-orbit services (December 2015; GEO: geostationary earth orbit; IGSO: inclined geo-synchronous orbit, MEO: medium earth orbit; FOC: full operational capability; IOV: in-orbit validation).

BDS				Galileo			
SVN	PRN	Type	Launched	SVN	PRN	Type	Launched
C003	C01	GEO	17 January 2010	E208	E08	FOC	17 December 2015
C004	C02	GEO	25 October 2012	E209	E09	FOC	17 December 2015
C004	C03	GEO	2 June 2010	E101	E11	IOV	21 October 2011
C006	C04	GEO	1 November 2010	E102	E12	IOV	21 October 2011
C011	C05	GEO	25 February 2012	E202	E14	FOC	22 August 2014
C005	C06	IGSO	1 August 2010	E201	E18	FOC	22 August 2014
C007	C07	IGSO	18 December 2010	E103	E19	IOV	12 October 2012
C008	C08	IGSO	10 April 2011	E104	E20	IOV	12 October 2012
C009	C09	IGSO	27 July 2011	E204	E22	FOC	27 May 2015
C010	C10	IGSO	2 December 2011	E205	E24	FOC	11 September 2015
C012	C11	MEO	30 April 2012	E203	E26	FOC	27 May 2015
C013	C12	MEO	30 April 2012	E206	E30	FOC	11 September 2015
C015	C14	MEO	19 September 2012				

**Table 2:** Summary of dynamic model.

Integration step-size	300 s
Geopotential (static)	EGM (Earth gravitational model) 2008 [32] up to degree and order 12
Solid earth tides	IERS (International Earth rotation and Reference systems Service) Conventions 2010 (Sun and Moon) [33]
Solid earth pole tides	IERS Conventions 2010
Relativistic effects	IERS Conventions 2010
Third-body	JPL (Jet Propulsion Laboratory) DE405 ephemeris used (Sun, Moon, Jupiter, Venus, Mars, Mercury, Uranus, Neptune, Saturn, Pluto, and Charon as point mass) [34]
Solar radiation pressure model	ECOM (Empirical Center for Orbit Determination in Europe Orbit Model)
Earth radiation model Numerical integration	GPS/GLONASS applied Runge–Kutta–Fehlberg method and Adams–Bashforth and –Moulton method [35]
Antenna thrust	Not applied

**Table 3:** Means and standard deviations (STDs) of weighted root-mean-square (WRMS) and median residuals of the selected fitted arc length (GPS: 40–45 h; GLONASS: 40–45 h; Galileo: 42–48 h; BeiDou inclined geo-synchronous orbit (IGSO): 40–50 h; BeiDou medium earth orbit (MEO): 42–54 h; unit: mm).

ECOM			GPS		GLONASS		Galileo		BDS (IGSO/MEO)	
			Mean	STD	Mean	STD	Mean	STD	Mean	STD
5	24 h	WRMS	45.9	10.8	76.0	23.0	111.4	33.0	101.4/118.4	33.9/48.9
		Median	37.0	9.1	61.9	15.2	105.4	35.7	94.6/109.4	32.6/50.5
	6 h	WRMS	15.9	3.3	24.1	6.4	44.7	10.8	39.3/39.9	13.2/15.4
		Median	13.8	2.7	20.9	4.8	39.6	11.7	37.3/38.4	13.4/16.1
9	24 h	WRMS	47.1	12.6	69.6	21.1	95.3	28.6	100.6/123.6	31.5/51.8
		Median	38.2	11.8	57.4	13.0	86.3	28.1	94.3/114.4	31.0/53.4
	6 h	WRMS	14.4	3.0	20.2	3.9	33.2	7.3	40.9/40.2	13.7/16.1
		Median	12.6	2.3	18.3	3.4	31.7	7.2	39.5/38.6	15.0/16.2

**Table 4:** The effects of Helmert rotational parameters on orbits at the nominal altitude of each navigation systems in the selected fitted arc length intervals (GPS: 40–45 h; GLONASS: 36–45 h; Galileo: 42–48 h; BeiDou inclined geo-synchronous orbit (IGSO): 42–48 h; BeiDou medium earth orbit (MEO): 39–54 h; BD-I/M represent BeiDou-IGSO/MEO, respectively; unit: mm).

		ECOM-5				ECOM-9			
		24 h		6 h		24 h		6 h	
		Mean	STD	Mean	STD	Mean	STD	Mean	STD
GPS	$R_{X}$	0.3	8.2	2.3	11.5	0.6	8.6	1.0	11.8
	$R_{Y}$	−2.4	8.1	−3.6	11.5	−2.0	8.5	−5.6	11.5
	$R_{Z}$	−3.8	23.4	−2.1	9.9	17.9	30.1	7.3	10.7
GLO	$R_{X}$	−1.4	12.8	−0.8	18.1	0.3	11.4	−0.2	13.9
	$R_{Y}$	1.6	9.4	1.1	19.9	1.7	9.2	0.5	16.7
	$R_{Z}$	14.7	34.0	16.2	20.3	17.4	33.4	19.4	12.8
GAL	$R_{X}$	−0.3	23.5	22.9	43.4	−2.9	27.4	11.0	36.3
	$R_{Y}$	2.0	24.3	27.5	53.4	7.0	26.9	6.4	45.7
	$R_{Z}$	−59.5	81.2	−64.3	69.5	−2.9	81.6	−39.9	50.3
BD-I	$R_{X}$	−2.8	49.9	−5.6	87.5	−7.3	50.7	−7.1	75.3
	$R_{Y}$	8.7	96.2	−19.0	122.7	5.3	83.7	−13.1	119.3
	$R_{Z}$	20.2	146.6	11.3	169.5	12.7	169.2	20.2	204.8
BD-M	$R_{X}$	−3.5	38.2	12.1	72.3	−4.3	35.3	−13.5	70.8
	$R_{Y}$	5.8	37.6	3.7	72.0	5.4	35.7	0.9	67.1
	$R_{Z}$	−22.4	155.3	−18.1	101.5	−23.0	169.8	−12.7	102.4

## Supplementary Materials

The following are available online at http://www.mdpi.com/1424-8220/18/2/477/s1: Figure S1: Means and standard deviations (STDs) for orbital scale (upper plane), translational offsets (middle plane) and rotational offsets (lower plane) of Helmert parameters for GPS. Results for reduced Empirical Center for Orbit Determination in Europe Orbit Model (ECOM-5) are shown in the left and for nine-parameter (ECOM-9) in the right plane. Figure S2: Means and standard deviations (STDs) for orbital scale (upper plane), translational offsets (middle plane) and rotational offsets (lower plane) of Helmert parameters for GLONASS. Results for reduced Empirical Center for Orbit Determination in Europe Orbit Model (ECOM-5) are shown in the left and for nine-parameter (ECOM-9) in the right plane. Figure S3: Means and standard deviations (STDs) for orbital scale (upper plane), translational offsets (middle plane) and rotational offsets (lower plane) of Helmert parameters for Galileo. Results for reduced Empirical Center for Orbit Determination in Europe Orbit Model (ECOM-5) are shown in the left and for nine-parameter (ECOM-9) in the right plane. Figure S4: Means and standard deviations (STDs) for orbital scale (upper plane), translational offsets (middle plane) and rotational offsets (lower plane) of Helmert parameters for BeiDou-IGSO. Results for reduced Empirical Center for Orbit Determination in Europe Orbit Model (ECOM-5) are shown in the left and for nine-parameter (ECOM-9) in the right plane. Figure S5: Means and standard deviations (STDs) for orbital scale (upper plane), translational offsets (middle plane) and rotational offsets (lower plane) of Helmert parameters for BeiDou-MEO. Results for reduced Empirical Center for Orbit Determination in Europe Orbit Model (ECOM-5) are shown in the left and for nine-parameter (ECOM-9) in the right plane. Table S1: The effects of Helmert transformation parameters on orbits at the nominal altitude of GPS in the selected fitted arc length intervals (40–45 h; Unit: mm; ECOM: Empirical Center for Orbit Determination in Europe Orbit Model). Table S2: The effects of Helmert transformation parameters on orbits at the nominal altitude of GLONASS in the selected fitted arc length intervals (36–45 h; Unit: mm; ECOM: Empirical Center for Orbit Determination in Europe Orbit Model). Table S3: The effects of Helmert transformation parameters on orbits at the nominal altitude of Galileo in the selected fitted arc length intervals (42–48 h; Unit: mm; ECOM: Empirical Center for Orbit Determination in Europe Orbit Model). Table S4: The effects of Helmert transformation parameters on orbits at the nominal altitude of BeiDou-IGSO in the selected fitted arc length intervals (42–48 h; Unit: mm; ECOM: Empirical Center for Orbit Determination in Europe Orbit Model). Table S5: The effects of Helmert transformation parameters on orbits at the nominal altitude of BeiDou-MEO in the selected fitted arc length intervals (39–54 h; Unit: mm; ECOM: Empirical Center for Orbit Determination in Europe Orbit Model).
