# Supplementary material for: Comparison of Ultra-Rapid Orbit Prediction Strategies for GPS, GLONASS, Galileo and BeiDou
Source: Sensors (Basel). 2018 Feb 6;18(2):477. doi: 10.3390/s18020477 (PMC5856057; doi:10.3390/s18020477)
Supplement: Supplementary file 1 [file sensors-18-00477-s001.pdf]

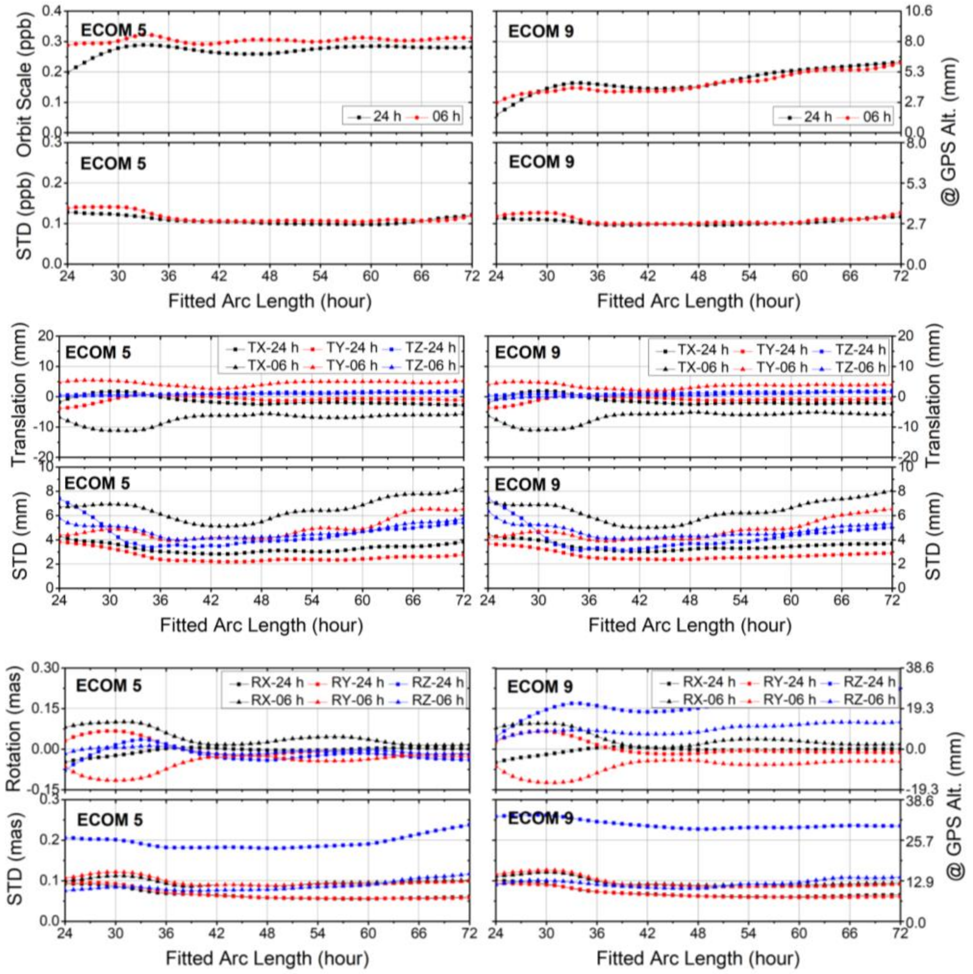

Figure S1. Means and standard deviations (STDs) for orbital scale (**upper** plane), translational offsets (**middle** plane) and rotational offsets (**lower** plane) of Helmert parameters for GPS. Results for reduced Empirical Center for Orbit Determination in Europe Orbit Model (ECOM-5) are shown in the left and for nine-parameter (ECOM-9) in the right plane.

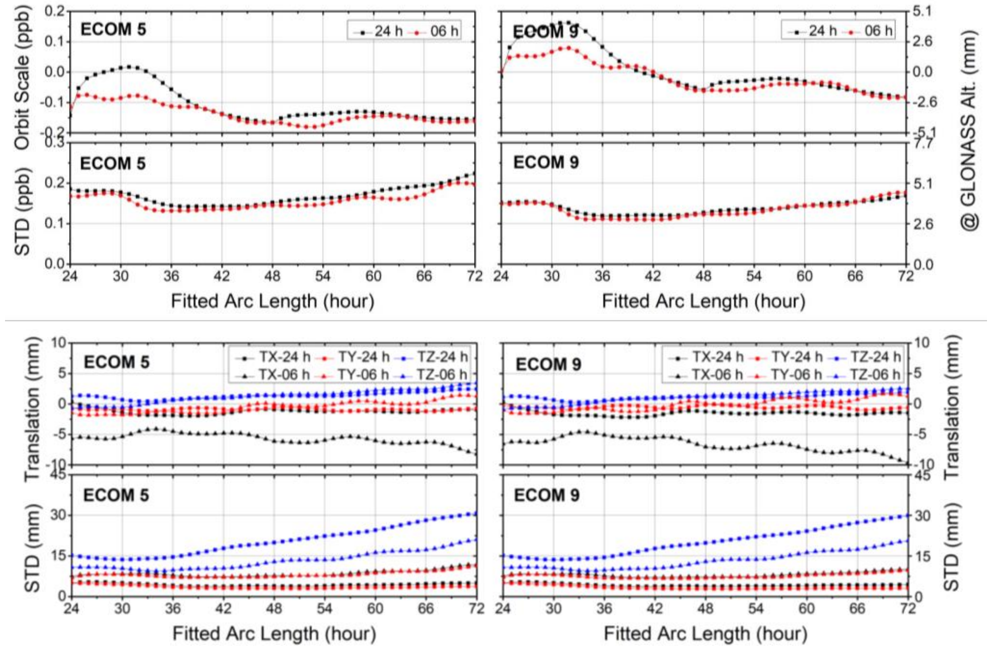

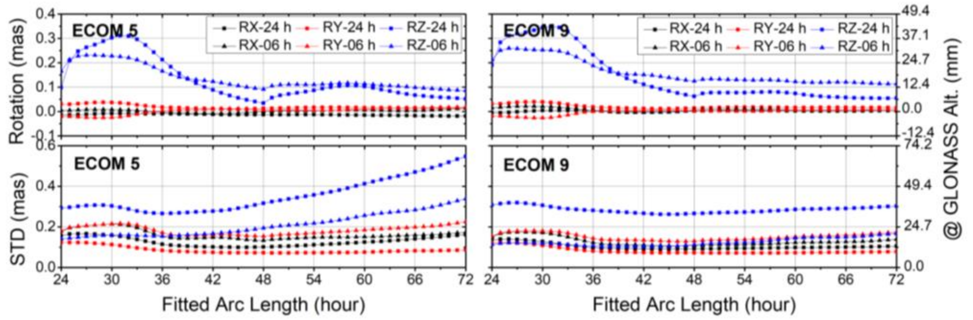

Figure S2. Means and standard deviations (STDs) for orbital scale (**upper** plane), translational offsets (**middle** plane) and rotational offsets (**lower** plane) of Helmert parameters for GLONASS. Results for reduced Empirical Center for Orbit Determination in Europe Orbit Model (ECOM-5) are shown in the left and for nine-parameter (ECOM-9) in the right plane.

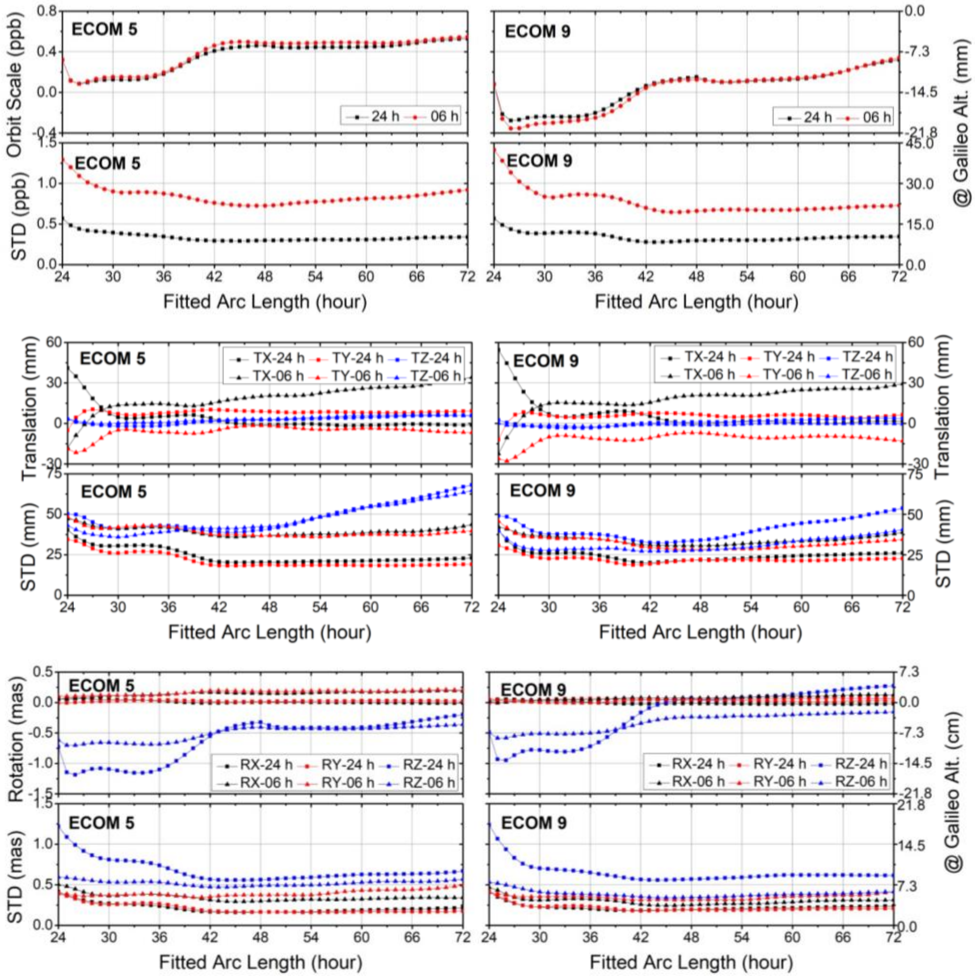

Figure S3. Means and standard deviations (STDs) for orbital scale (**upper** plane), translational offsets (**middle** plane) and rotational offsets (**lower** plane) of Helmert parameters for Galileo. Results for reduced Empirical Center for Orbit Determination in Europe Orbit Model (ECOM-5) are shown in the left and for nine-parameter (ECOM-9) in the right plane.

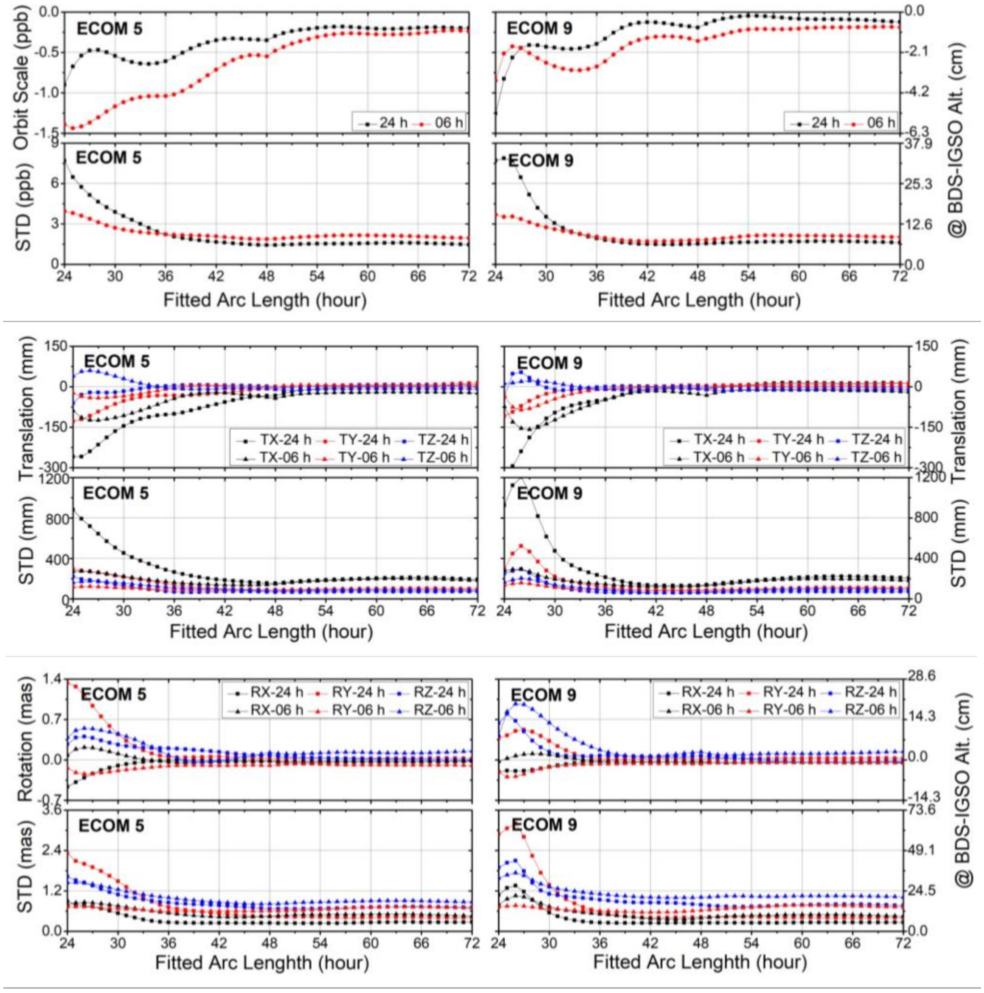

Figure S4. Means and standard deviations (STDs) for orbital scale (**upper** plane), translational offsets (**middle** plane) and rotational offsets (**lower** plane) of Helmert parameters for BeiDou-IGSO. Results for reduced Empirical Center for Orbit Determination in Europe Orbit Model (ECOM-5) are shown in the left and for nine-parameter (ECOM-9) in the right plane.

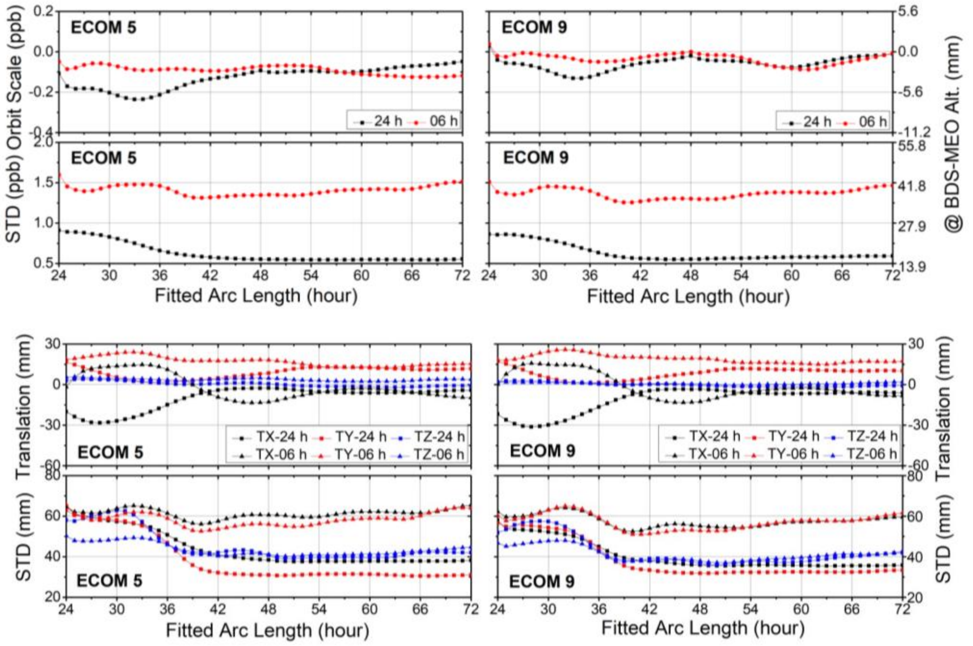

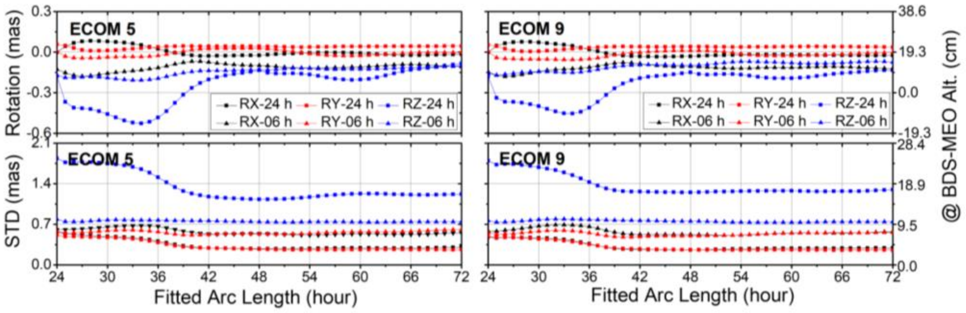

Figure S5. Means and standard deviations (STDs) for orbital scale (**upper** plane), translational offsets (**middle** plane) and rotational offsets (**lower** plane) of Helmert parameters for BeiDou-MEO. Results for reduced Empirical Center for Orbit Determination in Europe Orbit Model (ECOM-5) are shown in the left and for nine-parameter (ECOM-9) in the right plane.

Table S1. The effects of Helmert transformation parameters on orbits at the nominal altitude of GPS in the selected fitted arc length intervals (40–45 h; Unit: mm; ECOM: Empirical Center for Orbit Determination in Europe Orbit Model).

		**ECOM-5**				**ECOM-9**			
		**24 h**		**06 h**		**24 h**		**06 h**	
		**Mean**	**STD**	**Mean**	**STD**	**Mean**	**STD**	**Mean**	**STD**
	SC	7.0	2.8	7.9	2.8	3.9	2.6	3.6	2.6
	TX	−1.8	2.8	−6.3	5.2	−1.8	3.0	−5.7	5.0
	TY	−0.1	2.2	2.8	4.2	−0.4	2.4	2.2	4.1
GPS	TZ	0.9	3.5	0.8	4.1	1.0	3.3	0.5	4.2
	RX	0.3	8.2	2.3	11.5	0.6	8.6	1.0	11.8
	RY	−2.4	8.1	−3.6	11.5	−2.0	8.5	−5.6	11.5
	RZ	−3.8	23.4	−2.1	9.9	17.9	30.1	7.3	10.7

Table S2. The effects of Helmert transformation parameters on orbits at the nominal altitude of GLONASS in the selected fitted arc length intervals (36–45 h; Unit: mm; ECOM: Empirical Center for Orbit Determination in Europe Orbit Model).

		**ECOM-5**				**ECOM-9**			
		**24 h**		**06 h**		**24 h**		**06 h**	
		**Mean**	**STD**	**Mean**	**STD**	**Mean**	**STD**	**Mean**	**STD**
	SC	−3.0	3.6	−3.4	3.4	0.3	3.1	0.0	2.8
	TX	−1.7	3.8	−4.8	7.5	−1.9	3.8	−5.5	7.2
	TY	−0.8	3.2	−1.2	7.1	−0.5	3.0	−0.8	6.7
GLONASS	TZ	1.0	16.9	0.9	10.4	0.9	16.7	0.8	10.4
	RX	−1.4	12.8	−0.8	18.1	0.3	11.4	−0.2	13.9
	RY	1.6	9.4	1.1	19.9	1.7	9.2	0.5	16.7
	RZ	14.7	34.0	16.2	20.3	17.4	33.4	19.4	12.8

Table S3. The effects of Helmert transformation parameters on orbits at the nominal altitude of Galileo in the selected fitted arc length intervals (42–48 h; Unit: mm; ECOM: Empirical Center for Orbit Determination in Europe Orbit Model).

		**ECOM-5**				**ECOM-9**			
		**24 h**		**06 h**		**24 h**		**06 h**	
		**Mean**	**STD**	**Mean**	**STD**	**Mean**	**STD**	**Mean**	**STD**
Galileo	SC	13.3	8.8	14.6	22.0	3.5	8.5	3.0	19.8
	TX	−0.0	20.3	18.7	36.5	1.9	21.0	19.0	30.7
	TY	9.3	18.4	−2.4	37.1	7.2	20.7	−8.1	28.7
	TZ	2.4	39.5	2.8	41.8	0.7	33.0	−0.8	27.4
	RX	−0.3	23.5	22.9	43.4	−2.9	27.4	11.0	36.3
	RY	2.0	24.3	27.5	53.4	7.0	26.9	6.4	45.7
	RZ	−59.5	81.2	−64.3	69.5	−2.9	81.6	−39.9	50.3

Table S4. The effects of Helmert transformation parameters on orbits at the nominal altitude of BeiDou-IGSO in the selected fitted arc length intervals (42–48 h; Unit: mm; ECOM: Empirical Center for Orbit Determination in Europe Orbit Model).

		**ECOM-5**				**ECOM-9**			
		**24 h**		**06 h**		**24 h**		**06 h**	
		**Mean**	**STD**	**Mean**	**STD**	**Mean**	**STD**	**Mean**	**STD**
BeiDou-IGSO	SC	−14.3	64.2	−6.5	62.6	−24.8	81.9	−13.4	73.2
	TX	−41.4	166.2	−31.4	139.4	−6.4	132.5	−21.8	123.6
	TY	−12.4	92.4	−0.0	87.1	3.4	81.3	−4.1	85.6
	TZ	3.3	70.8	−6.6	84.9	0.6	60.8	−7.7	62.9
	RX	−2.8	49.9	−5.6	87.5	−7.3	50.7	−7.1	75.3
	RY	8.7	96.2	−19.0	122.7	5.3	83.7	−13.1	119.3
	RZ	20.2	146.6	11.3	169.5	12.7	169.2	20.2	204.8

Table S5. The effects of Helmert transformation parameters on orbits at the nominal altitude of BeiDou-MEO in the selected fitted arc length intervals (39–54 h; Unit: mm; ECOM: Empirical Center for Orbit Determination in Europe Orbit Model).

		**ECOM-5**				**ECOM-9**			
		**24 h**		**06 h**		**24 h**		**06 h**	
		**Mean**	**STD**	**Mean**	**STD**	**Mean**	**STD**	**Mean**	**STD**
BeiDou-MEO	SC	−3.2	15.7	−2.2	37.3	−1.3	15.6	−0.6	36.1
	TX	−4.1	39.4	−9.2	59.2	−4.8	36.7	−9.1	54.6
	TY	7.9	31.7	17.0	54.8	7.8	32.7	18.9	52.7
	TZ	0.3	41.0	4.0	41.0	−0.4	37.6	0.3	38.0
	RX	−2.7	38.6	−12.7	72.5	−3.7	35.9	−13.8	70.9
	RY	5.6	37.3	2.5	71.7	5.4	35.7	0.2	67.3
	RZ	−24.7	157.4	−17.8	101.3	−24.4	170.7	−12.3	102.0
